# Social Anxiety in 15–19 Year Adolescents in Association with Their Subjective Evaluation of Mental and Physical Health

**DOI:** 10.3390/children8090737

**Published:** 2021-08-26

**Authors:** Ingrida Pilkionienė, Giedrė Širvinskienė, Nida Žemaitienė, Jolita Jonynienė

**Affiliations:** 1 Department of Health Psychology, Lithuanian University of Health Sciences, Tilžės 18, LT-47181 Kaunas, Lithuania; giedre.sirvinskiene@lsmuni.lt (G.Š.); nida.zemaitiene@lsmuni.lt (N.Ž.); jolita.jonyniene@lsmuni.lt (J.J.); 2 Health Research Institute, Lithuanian University of Health Sciences, Tilžės 18, LT-47181 Kaunas, Lithuania

**Keywords:** adolescents, social anxiety, mental health, physical health

## Abstract

Background: Studies show that social anxiety in adolescence have negative impact on quality of life. The study evaluates social anxiety links with mental and physical health factors in adolescents aged 15–19 years. Methods: The research was performed in 2018 in secondary schools in Lithuania and included 1722 participants (46.1% males and 53.9% females). The social anxiety was assessed using Social Anxiety and Avoidance Scale for Adolescents. The main results were obtained using univariate and multivariate logistic regression analysis. Results: Total of 58.5% of adolescents were characterized by high social anxiety and 14.7% by high avoidance. Females more often were characterized by high anxiety compared to males. Multivariate logistic regression revealed that good mental health was a significant protective factor against high SA in adolescents. For females, high anxiety and avoidance were associated with living with both parents, for males, high anxiety was linked with mother’s university education. Very common stomach and abdominal pain in females as well as severe and very common stomach or abdominal pain in males, increase the risk of major social anxiety. Conclusions: High social anxiety were more prevalent between females than males and was linked with various well-being and health aspects in adolescents.

## 1. Introduction

Social anxiety (SA) is described as a strong and persistent anxiety in a variety of social situations which might lead to other mental health disorders [1]. When SA reaches the level when a child’s functioning is impaired (child experiences persistent anxiety in a number of situations including inherent caution in dealing with strangers, social fear, anxiety in facing a new, unfamiliar, or socially dangerous situations), it is classified as Social Anxiety Disorder [2]. Various social fears (especially related to being evaluated by others) usually begin or intensify in late childhood or early adolescence [3].

In the past decade, the prevalence of SA varied greatly across the clinical sample and general population but remained similar despite of country of origin. Various research declared that SA prevalence counted 7 to 13% for the whole population [4]. The prevalence of social anxiety disorder in children and adolescents ranges from 1.8% till 9% or 11.7% [5,6,7] and 51% of adolescents dealt with social fear in at least one type of situations (which usually does not grow into more serious disorders) [3]. New study reported that prevalence of social anxiety to be significantly higher than previously reported, with more than 1 in 3 (36%) respondents meeting the threshold criteria for having Social Anxiety Disorder (SAD) [8].

Results of empirical studies showed that high SA was associated with a variety of mental health difficulties. It was indicated that SA disorder in adolescence predicted higher depression level [9]. Van Zalk and Tillfors (2017) confirmed the connection between SA and depressive symptoms [10]. The study by Klemanski and colleagues (2017) explained that adolescents with high levels of SA and depressive symptoms were characterized by decreased understanding and expression of emotions as well as emotion management strategies [11]. SA was also associated with adolescent suicide risk [12,13]. 

Poorer mental health is known to be associated more frequently with physical health. Studies showed that SA correlated with chronic migraine (irrespective of demographic characteristics) and pain intensity [14], complaints about any other pain in one’s body [15] as well as low sleep quality and high rates in body fat [16]. Means-Christensen et al. (2008) found that respondents with more severe pain symptoms reported lower mental health functioning and higher scores on severity measures of depression, social anxiety, and post-traumatic stress disorder [17]. The available research seems to suggest that somatic symptoms are highly prevalent among children and adolescents with anxiety disorders and are associated with greater anxiety severity and impairment [18] and are consistently associated with anxiety and depressive symptoms [19]. Adolescent psychosomatics has been analyzed for many years [20,21] and linked to various physical pains [22,23].

Studies also confirmed that SA in adolescence had a significant negative impact on a number of factors of adolescent’ quality of life, for example: physical and mental health, social relationships, leisure time, etc. [24,25].

In the scientific literature, the links between SA and sociodemographic factors are widely analyzed. Studies showed that girls, more often than boys, experienced SA [6,26,27,28]. In addition, SA was likely to disrupt girls’ social activities more than boys’ [29]. However, several research studies in Finland [30] and Pakistan [24] revealed no significant differences regarding adolescents’ sex or demonstrated even controversial results. Cakin Memik and colleagues (2010) found that boys demonstrated higher SA and negative assessment rates than girls. Researchers also suggested that the research method might have considerable implications on the extent to which and how SA was revealed [31]. Research has showed correlation between SA and age. This might depend on the specific age range chosen by researchers. Some studies declared that older adolescents (14–16-year-olds) reported higher SA in comparison to younger adolescents (12–13-year-olds) [25]. Moreover, the likelihood of SA disorder was significantly higher for older adolescents [5]. In the studies with wider age ranges, La Greca (1998) [29] found that SA scores were higher in high school than in primary school students. Farooq and co-authors (2017) suggested that SA was not related to age in a group of 14- to 17-year olds [25].

In scientific literature, a number of research confirmed the importance of family life on children’s and adolescents’ mental health, e.g., harmonious family relationships [32], democratic parenting style [33,34]. Mohammadi et al. (2020) found a lower prevalence of SA among individuals with higher parental education [5]. Nevertheless, there are a number of studies which claimed for limited insignificant correlation between SA and family structure, parental education or even profession-related activities [35,36].

To sum up, high SA levels put adolescents at risk of developing long-lasting mental and physical health symptoms. Therefore, it is particularly important to alert parents, teachers, and clinicians with deeper understanding about SA and its synergistic relationship with various factors so that they could identify the SA signs as early as possible. [37]. So, it is important to have a deeper understanding of adolescents’ social anxiety and the synergistic relationship between various factors.

The aim of this study is to analyze the associations between SA, mental, and physical health in 15- to 19-year-old adolescents (females and males).

## 2. Materials and Methods

### 2.1. Research Procedure and Sample

Research was performed from March to June, 2018 in Lithuanian secondary schools. The research was approved by the Bioethics Center of the Lithuanian University of Health Sciences No. BEC-VS (D)-36 (29 November 2017). Prior to the study, written school administration consents were obtained and informed consents were prepared for parents/guardians and study participants (students). Inclusion criteria: high school students aged 15 to 19 years, agreement provided by students and parents to participate in the study. Researchers believe that all the students (even with mild learning disabilities) were able to read and understand the research questionnaire. If needed, special pedagogues, who usually help pupils with special needs, or instructed researchers assisted the students to read the questions. Exclusion criteria: age lower than 15 years and higher than 19 years, and disagreement of participation in the study of student or/and parents/ guardians.

The study was completed by trained researchers who had specific guidelines related to how to collect data. Researchers met the students (research participants) at their school classrooms. Students were invited to complete anonymous questionnaire. The anonymity of the subjects was ensured. The time to complete the questionnaire was equal to one lesson (approximately 45 min). 

Adolescents aged 15 to 19 years (grade 9 to 12) participated in the study. In order to statistically reliably estimate the probability of SA in the population, minimum sample volumes were calculated and a representative sample was formed. As a result of that, the final sample in this research study included 1722 adolescents (response rate 89.45%), 794 male (46.1%), and 928 females (53.9%) aged 15 to 19 years (average age 16.6 years, SD = 1.05) (see Table 1).

### 2.2. Outcome Measures

Social anxiety was measured using Social Anxiety and Avoidance Scale for Adolescents-SAASA [37]. The scale consisted of two subscales, measuring (1) anxiety and (2) avoidance experienced. Adolescents were provided with 34 social situations for each subscale (same situations to measure anxiety and avoidance). For stating how strong anxiety they feel and how often they avoid these social situations, participants were invited to rate their answers from 1 “never” to 5 “almost always”. The total score, for each subscale, may range from 34 and 170 points. Examples of social situations: “Reading aloud in front of the class”; “Writing while being observed”; “Expressing disagreement or disapproval to a colleague, I don’t know very well”; “Going to a party given by a colleague”; “In a bus or train, sitting in front of other people”; “Saying “no” to a colleague that has asked me to do something I don’t want to do”; “Expressing my feelings to the person I like”; “Talking to someone I don’t know very well”. The higher scores indicate higher levels of anxiety and avoidance of social situations [37,38]. Subscale items are summed so that higher scores reflect greater social anxiety [39,40]. Scores exceeding the average scores of the corresponding Anxiety and Avoidance scales by 1 standard deviation were considered as high social anxiety and social avoidance, following the study [41]. In our study the average score for SA was equal to 2.27 (SD = 0.72; min = 1, max = 4.91), for avoidance it was equal to 2.20 (SD = 0.69, min = 1, max = 4.91). The resulting cut-offs were thus equal to 2.99 and 2.90 for the Anxiety and Avoidance scales, correspondingly. Finally, we chose 2.99 and 2.99 as the cut-offs for both scales. The cut-off values vary across the different studies [29,42]. For example, for 22 items in a 5-point Likert scale, a 55 cut-of-point suggested as a good score to distinguish adolescent with and without social anxiety disorder in a community sample (the cut-off equals to the average score of 2 obtained by 22 * 5/55 = 2) [42]; other authors used 18 anxiety items of a 5-point Likert scale and suggested a cut-off point of 1.8 (equals to the average score of 1.8 obtained by 18 * 5/50 = 1.8) [24,29].

Our approach of cut-off definition allowed us to form groups of highly expressed social anxiety and social avoidance. High levels of social anxiety put the adolescent at risk of developing more severe long-range problems [37]. This study aims to gain a deeper understanding of adolescents’ high social anxiety and its links with important protection and risk factors.

Adolescents were divided into groups based on the self-assessed SA scores (SA severity):

Anxiety groups—(1) *low-level anxiety* (anxiety scores lower than 2.99) and (2) *high-level anxiety*, (anxiety scores higher than 3.00);

Avoidance groups—(1) *low-level avoidance* (avoidance score lower than 2.99) and (2) *high-level avoidance* (avoidance scores higher than 3.00).

The internal consistency for SAASA with the Lithuanian sample was indicated as high (Cronbach alpha for Anxiety subscale equal to 0.95, Avoidance subscale—0.93). The authors of the scale indicated Cronbach alphas accordingly equal to 0.91 and 0.87 [43]. 

In this study, several independent variables were included to explore if they might increase or decrease the likelihood that a specific adolescent is included into one or another group. 

### 2.3. Independent Variables

Subjective evaluation of mental health was assessed using Mental Health Continuum-Short form (MHC-SF, 2009) [44] which measured emotional, social, and psychological well-being. In the original validation study, Cronbach alpha for the MHC-SF scale was found to be equal to 0.89. The maximum score of MHC-SF was equal to 6 (which means “every day”), the minimum—1 (equal to “never”). The authors of the original scale pointed out that the higher the score, the better the subjective evaluation of mental health. In this Lithuanian study, factor analysis based on rotation method (Varimax with Kaiser Normalization) was completed (KMO = 0.911; *p* < 0.001). As a result of that, mental health scale (Cronbach’s alpha 0.88) was divided by authors of this study into two factors:

1. Psychological well-being—9 statements (Coefficients of variables from 0.720 to 0.545), e.g., During the past month, you felt: “Happy”, “Interested in life”, “Confident to be able to have and express your ideas or opinions” (Cronbach’s alpha 0.85).
2. Social well-being—5 statements (Coefficients of variables from 0.827 to 0.608), e.g., “During the past month, you felt that you belonged to a community (such as a class, school, or other social group), that someone contributed or served society” (Cronbach’s alpha 0.80).

Subjective evaluation of physical health. Researchers decided to add items about physical health based on the Health Behaviour in School-aged Children study (known as HBSC) [45], specifically Health Symptom Checklist [46]. The original HBSC Health Symptom Checklist consists of eight items (four physical and four psychological symptoms). Four items regarding adolescents’ subjective evaluation of their physical health symptoms (three items about physical pain and one about sleep) were included in the questionnaire. The other four item were rejected because they correlated with the psychological well-being factor. Participants were asked about physical health symptoms during the last 12 months: (1) headache, (2) stomach or abdominal pain, (3) back pain, (4) lack of sleep and rest. Answers from 1—“I felt this rarely or never” to 5—“almost every day”. When analyzing the data, the variable was recoded into: (1) rare (“rarely or never”); (2) often (“almost every month”, “almost every week”); (3) very often (“more than once a week” “almost every day”).

Sociodemographic characteristics were measured including (1) sex (male or female), (2) age (in years), (3) family structure with two options: two parents or a single parent, (4) mother’s and father’s education. Participants could choose from several answer options and in the analysis dichotomous variable was created including university or lower than university education (in data analysis, education variable was transformed to dichotomous variable “university vs. lower than university”). In this study we did not include the answers of 228; the respondents “who do not know and do not remember” the education of their parents.

### 2.4. Statistical Analysis

For statistical data analysis, IBM SPSS Statistical package, 24 version was employed. First, two independent samples (groups by low vs. high anxiety and then low vs. high avoidance) were compared with the non-parametric Mann–Whitney U test (because the data distributions did not meet the normality assumptions). The normality of distributions was tested with Kolmogorov–Smirnov or Shapiro–Wilk tests. The Chi-square test was used to determine a statistically significant difference in categorical variables between social anxiety/avoidance groups. With the purpose of deeper understanding about the constructs explored, factor analysis was applied for extracting the psychological and social well-being factors. Finally, univariate and multivariate binary logistic regression was used to calculate the odds ratio (OR) of belonging to a group of high-level anxiety and high-level avoidance and build general models consisting of more risk factors (multivariate logistics regression). All the statistical analysis was completed for the male and female samples separately. Statistical significance level *p* was set at *p* < 0.05.

## 3. Results

### 3.1. Prevalence of Social Anxiety and Avoidance

The statistical data analysis revealed that 58.5% (n = 971) of adolescents were characterized by high anxiety. High avoidance was determined to be 14.7% of the research sample (n = 252) with most adolescents (85%) low on avoidance in social situations. 

SA results in different groups regarding sociodemographics are presented in Table 2. Descriptive analysis showed that more females were included in the group of high- level anxiety, and males were more likely to be included in the group of weakly expressed anxiety. No statistically significant association between avoidance and age was found, but avoidance was found to be more significant among older adolescents. The results of this study also suggested that high anxiety was more common among adolescents from single-parent families and whose parents were less educated, but adolescent avoidance was related only to the father’s education. 

### 3.2. Predictors of SA and Avoidance in Females: Univariate Logistic Regression Analysis

In that females’ “social anxiety (anxiety and avoidance) scores were significantly higher than males” (see Table 2), the following regression analysis was completed for females and males separately.

Univariate logistic regression analysis showed that females’ psychological and social well-being reduced the possibility that they would be higher on the anxiety and avoidance scores (see Table 3). The better psychological and social well-being, the less possibility for females’ high anxiety and avoidance. Univariate analysis also revealed that avoidance was significantly connected to physical health symptoms and well-being during the last 12 months. Females with severe headaches, stomach or abdominal pain, lack of sleep and rest had higher avoidance scores. For female sample, females’ age, family composition, and parental education were found not to be related to SA (anxiety and avoidance).

### 3.3. Predictors of SA and Avoidance in Males: Univariate Logistic Regression Analysis

Univariate logistic regression analysis showed that opportunity for male adolescents with higher psychological and social wellbeing scores to have high SA rates was lower (see Table 4). For males, maternal university education level as well as having physical health symptoms (headache, stomach, and abdominal pain, back pain, lack of sleep and rest) during the last 12 months predicted lower SA. The more severe headache, stomach and abdominal pain in adolescents, the higher the SA scores were. It was also found that males’ age and family structure variables were not related to high SA.

### 3.4. Predictors of High SA (Anxiety and Avoidance Groups): Multivariate Logistic Regression Analysis

**Figure 1:**
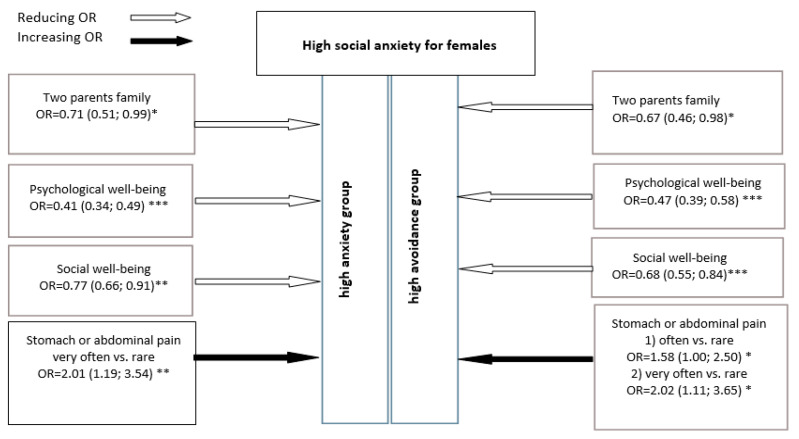
Multivariate logistic regression model for females: opportunity ratio (95% CI) to be high on SA in context of independent variables (* *p* < 0.05; ** *p* < 0.01; *** *p* < 0.001).

In the multivariate logistic regression analysis, mental health remained the protective factor for SA: the higher the females’ psychological and social wellbeing, the lower the SA and avoidance rates in a female sample. Furthermore, living with both parents (full family) was found as a protective factor for SA. Females with stomach or abdominal pain reflected to have a double opportunity to have higher SA scores (see Figure 1).

**Figure 2:**
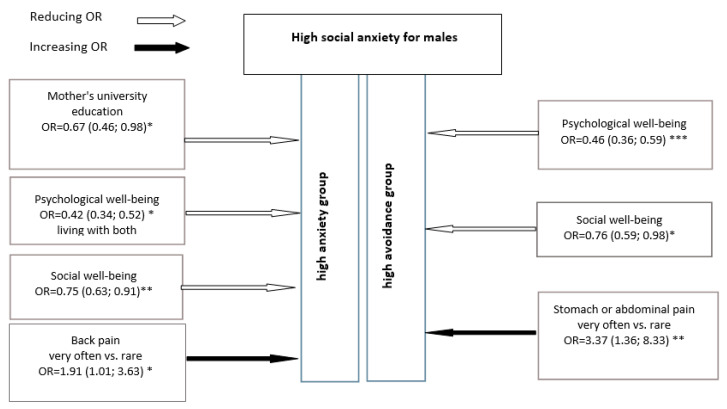
Multivariate logistic regression model for males: opportunity ratio (95% CI) to be high on SA in context of independent variables (* *p* < 0.05; ** *p* < 0.01; *** *p* < 0.001).

For males, mental health was also revealed as a protective factor for SA. The higher the males’ psychological and social well-being, the lower the SA and avoidance rates in a male sample. However, for comparing the research results for males and females, several significant differences were found. Males with back pain reflected in the research had a double opportunity to have higher SA scores; males with stomach and abdominal pain had a triple opportunity to have higher avoidance scores. Finally, maternal university education level was found as a significant protective factor for lower avoidance scores (this met the results of univariate regression analysis) (see Figure 2).

## 4. Discussion

This research study revealed some important SA associations with sociodemographic characteristics as sex, age, and family structure or educational level results showing that females scored higher on SA than males. Many other researchers proposed similar findings with girls experiencing SA more often [6,26,27,30,37,47]. Scientific literature explained increased vulnerability for anxiety disorders in women by the role of female reproductive hormones, differences in brain structures responsible for anxiety and panic related circuitry [48]. Garcia-Lopez et al. (2008) [27] added that despite of the fact that girls experienced more SA, social situations and circumstances were somewhat similar for both genders. We found that older adolescents scored higher on social avoidance, but not on anxiety. However, multivariate logistic regression suggested no significant association between SA and age neither in females nor in males group. Results of other research studies are controversial. Though Farooq and co-authors (2017) found no links between SA and age [24], others reported that SA intensified in early adolescence [27] or younger students [49]. Garcia-Lopez et al., (2005) revealed that SA scores decreased at the age of 16 years and then started increasing at 17 years [50]. Actually, in this study, adolescents were of similar age (mean age equal to 16.6 years) and this might be one of the reasons why age remained an insignificant factor for SA in regression models. Family structure was found to be related with SA as living with both parents was found as a protective factor for girls’ SA, but not males. The importance of family life on children’s and adolescents’ mental health is well documented in previous research, e.g., harmonious family relationships [32], democratic parenting style [33,34]. Other studies showed that maternal anxiety, stress, or even maternal control correlated with adolescents’ SA scores [51,52]. Possibly, females are more sensitive to family life and react to psychological-social changes intensively. Another sociodemographic characteristic—maternal education was found to be a protective factor for males SA. Other researchers shared controversary findings. Mohammadi et al. (2020) found a lower prevalence of SA among individuals with higher parental education [5]. Nevertheless, there are a number of studies that claimed for limited insignificant correlation between SA and family structure, parental education or even profession-related activities [35,36]. Regarding this study, it is challenging to interpret the specific finding (maternal education vs. males’ SA) in that a lot of adolescents had actually limited information about parental education. Therefore, additional studies are needed to explain this correlation carefully. 

Another part of analysis consisted of SA links with mental and physical health aspects. SA has been found to be linked with physical and mental health by other authors [53].

Our survey showed that the psychological and social well-being of adolescents remained an essential protective factor in reducing risk for high levels of anxiety and avoidance. According to Klemanski and colleagues (2017), adolescents with high social anxiety and depressive symptoms complained of decreased emotional understanding, expression, and decreased use of emotion management strategies [11]. The results of Lima and co-authors (2020) found that social anxiety was indirectly associated with depressive symptoms due to poor sleep quality. According to researchers, social anxiety may have an important role in adolescents’ physical and mental health [53]. We found the links of high levels of anxiety and avoidance with physical health complaints. In females persistent high social anxiety and stress were significantly associated with stomach and abdominal pain, for males, was associated with stomach, abdominal, and back pain. Similar links were found in the works of other authors. The findings of a study by Cunningham et al., (2015) suggest that children and adolescents with functional abdominal pain (FAP) have increased generalized anxiety, separation anxiety, and school dropout rates [15]. The results of a study by Means-Christensen et al. (2008) suggest that respondents who confirmed pain symptoms also complained of poorer mental health: they had higher rates of the severity of depression, social anxiety, and post-traumatic stress disorder [17]. Adolescent psychosomatics has been analyzed for many years [20,21] and links to various physical pains [22,23]. We may consider that stress experienced by adolescents in social situations causes psychosomatic symptoms.

Analyzing the expression of adolescent social anxiety, our study found that high anxiety was characteristic for more than a half of students and avoidance in social situations in about 15% subjects. Examining the reciprocal links between experiential avoidance and social anxiety among high school students, Japanese researchers found that a previous propensity for social anxiety later had a positive effect on slightly higher experiential avoidance at each time point and suggest that social anxiety may lead to avoidance [54]. Other researchers also point to avoidant behavior and its links to social anxiety. The findings of a study by Kashdan et al., (2010), found an association between avoidance and an increase in social anxiety (rather than depression or anger) over 3 months [55]. Thus, we can hypothesize that adolescents with severe anxiety may also experience increased avoidance in the future.

Therefore, it is essential both to identify SA and ensure that the adolescent receives the necessary help as early as possible before the problem is over and has reached the level of the disorder. It was observed that about 25 percent of adolescents with social anxiety disorder seek help [38] and that most people with social anxiety disorder do not seek psychological help [12]. It is vital to diagnose SA as early as possible and provide the necessary care, as this disorder can escalate into other serious problems such as alcohol and drug use, depression, suicidal behavior, and more [56]. Comorbidity, psychosomatic symptoms characterize SA, so parents, teachers, health care professionals need to pay attention when adolescents have complaints of psychological and physical well-being. For example, when they express intense anxiety in social situations or avoidance or noticing other mental health difficulties or physical health complaints (stomach, abdomen, back, headache, pain, sleep difficulties), as young people with social anxiety often do not seek professional help themselves [27]. Another area is adolescents’ psychoeducation-teaching adolescents how to deal with stressful and worrying social situations. As Tillfors and colleagues (2012) observe, it is very important to develop the personal qualities of adolescents to avoid encountering intimidating, unpleasant situations [57].

It is important to note several limitations of this study. One of the limitations was that only the adolescent questionnaire was conducted, and there was no possibility to include parental or teacher as respondents in the study. It turned out that a significant number of study participants were unaware of parental education, which made it difficult to assess in more detail the importance of parental education for adolescents’ social anxiety and avoidance. Limiting the adolescent survey increases the possibility of subjectivity because it does not include more objective information gathered from other informants and assessing social anxiety and avoidance symptoms. One more limitation is that we used questionnaire survey and did not make clinical assessment of anxiety. In the future, it would be important to include clinical assessment and objective health information collected by health professionals, such as data on adolescents’ physical health from health facilities (chronic illnesses, etc.), in studies that assess social anxiety, avoidance, and physical symptoms.

## 5. Conclusions

In this study for adolescents (female and male) social anxiety was related to subjective evaluation of physical and mental health. For females, living with both parents was a protective factor against SA, while for males, the mother’s university education remained a significant protective factor, but only in a group of high level anxiety. The likelihood of the occurrence of severe social anxiety doubled in females showing stomach or abdominal pain. In males, a common association of severe anxiety with back pain and severe anxiety with chest or abdominal pain was found.

## Figures and Tables

**Table 1:** Sociodemographic factors.

Demographic Characteristics	Number of Participants (Percentage)
*Sex*	
females	928 (53.9%)
males	794 (46.1%)
*Age*	
15–16	794 (46.1%)
17–19	928 (53.9%)
*Family composition*	
two parients families	1113 (64.4%)
single parent families	614 (35.6%)
*Education of mothers*	
lower than university	814 (47.1%)
university	408 (23.6%)
does not know/remember	307 (17.8%)
*Education of fathers*	
lower than university	855 (49.5%)
university	408 (23.6%)
does not know/remember	464 (26.9%)

**Table 2:** SA and sociodemographic factors.

Variables, n (%)	Social Anxiety					
	Low Anxiety	High Anxiety	χ^2^ (*p*)	Low Avoidance	High Avoidance	χ^2^ (*p*)
Sex						
*Male*	421 (54.1)	357 (45.9)	79.46	701 (88.7)	89 (11.3)	13.66
*Female*	297 (32.6)	614 (67.4)	(<0.001)	763 (82.4)	163 (17.6)	(<0.001)
Family structure						
*Two parents*	485 (44.3)	609 (55.7)	4.17	956 (86.4)	151 (13.6)	2.63
*Single-parent*	234 (39.2)	363 (60.8)	(0.041)	510 (83.5)	101 (16.5)	(0.105)
Mother‘s education						
*Other than university*	302 (37.9)	495 (62.3)	18.71	680 (84.0)	130 (16.0)	2.46
			(<0.001)			(0.117)
*University*	294 (49.5)	300 (50.5)		526 (86.9)	79 (13.1)	
Father‘s education						
*Other than university*	346 (41.6)	485 (58.4)	4.53	722 (84.8)	129 (15.2)	3.89
			(0.033)			(0.049)
*University*	195 (48.0)	211 (52.0)		362 (88.9)	45 (11.1)	
	Low anxiety	High anxiety	*p*	Low avoidance	High avoidance	*p*
Age in years						
median (min, max)	17 (15;19)	17 (15;19)	0.464	17 (15;19)	17 (15;19)	0.029
average range	853.22	836.31		846.59	917.35	

**Table 3:** Univariate logistic regression: the ratio of females’ odds ratio to be high on SA (anxiety and avoidance groups).

	High Anxiety		High Avoidance	
	OR (95% CI)	*p*	OR (95% CI)	*p*
*Age in years*	0.988 (0.868; 1.124)	0.851	1.132 (0.966; 1.326)	0.126
*Family structure*				
two parents vs. single-parent family	0.776 (0.581; 1.037)	0.086	0.817 (0.579; 1.152)	0.248
*Maternal education*				
university vs. non-university	0.760 (0.558; 1.037)	0.084	0.928 (0.632; 1.364)	0.704
*Paternal education*				
university vs. non-university	0.949 (0.663; 1.357)	0.773	0.882 (0.557; 1.395)	0.591
*Mental health:*				
(a) Psychological well-being	0.419 (0.350; 0.503)	<0.001	0.480 (0.397; 0.580)	<0.001
(b) Social well-being	0.801 (0.689; 0.932)	0.004	0.713 (0.590; 0.862)	<0.001
*Physical health symptoms*				
1. *Headache*				
(a) often vs. rare	1.308 (0.936; 1.827)	0.115	2.186 (1.337; 3.574)	0.002
(b) very often vs. rare	1.761 (1.199; 2.586)	0.004	2.426 (1.437; 4.096)	0.001
2. *Stomach and abdominal pain*				
(a) often vs. rare	1.306 (0.965; 1.769)	0.084	1.615 (1.067; 2.444)	0.024
(b) very often vs. rare	2.063 (1.271; 3.348)	0.003	2.501 (1.468; 4.261)	0.001
3. *Back pain*				
(a) often vs. rare	1.144 (0.845; 1.547)	0.384	1.358 (0.930; 1.982)	0.113
(b) very often vs. rare	1.326 (0.866; 2.030)	0.194	1.909 (1.181; 3.084)	0.008
4. *Lack of sleep and rest*				
(a) often vs. rare	1.585 (1.006; 2.496)	0.047	0.823 (0.421; 1.608)	0.568
(b) very often vs. rare	1.854 (1.209; 2.842)	0.005	1.978 (1.088; 3.596)	0.025

Note. OR—odds ratio (>1—increased likelihood, <1—reduced likelihood), CI—confidence interval 95 proc. *p* < 0.05.

**Table 4:** Univariate logistic regression: the ratio of males’ odds ratio to be high on SA (anxiety and avoidance groups).

	High Anxiety		High Avoidance	
	OR (95% CI)	*p*	OR (95% CI)	*p*
*Age in years*	0.903 (0.787; 1.037)	0.148	1.117 (0.903; 1.381)	0.309
*Family structure* two parents vs. single-parent family	0.925 (0.684; 1.251)	0.613	0.831 (0.524; 1.318)	0.431
*Maternal education* university vs. non-university	0.637 (0.464; 0.876)	0.006	0.738 (0.447; 1.216)	0.233
*Paternal education* university vs. non-university	0.793 (0.564; 1.116)	0.184	0.574 (0.314; 1.048)	0.071
*Mental health:*				
(a) Psychological well-being	0.450 (0.376; 0.540)	<0.001	0.451 (0.354; 0.574)	<0.001
(b) Social well-being	0.788 (0.679; 0.915)	0.002	0.744 (0.590; 0.937)	0.012
*Physical health symptoms*				
1. *Headache*				
(a) often vs. rare	1.572 (1.156; 2.137)	0.004	1.135 (0.696; 1.850)	0.613
(b) very often vs. rare	1.487 (0.922; 2.396)	0.103	1.970 (1.037; 3.743)	0.038
2. *Stomach and abdominal pain*				
(a) often vs. rare	1.411 (1.045; 1.905)	0.025	1.134 (0.705; 1.824)	0.605
(b) very often vs. rare	2.460 (1.216; 4.979)	0.012	3.557 (1.610; 7.861)	0.002
3. *Back pain*				
(a) often vs. rare	1.224 (0.899; 1.666)	0.198	0.994 (0.614; 1.608)	0.980
(b) very often vs. rare	1.831 (1.160; 2.891)	0.009	1.169 (0.592; 2.309)	0.654
4. *Lack of sleep and rest*				
(a) often vs. rare	1.379 (0.946; 2.011)	0.095	1.078 (0.576; 2.018)	0.814
(b) very often vs. rare	1.950 (1.349; 2.821)	<0.001	1.645 (0.921; 2.938)	0.093

Note. OR—odds ratio (>1—increased likelihood, <1—reduced likelihood), CI—confidence interval 95 proc. *p* < 0.05.

## Data Availability

The data presented in this study are not available for sharing.
